# Demographic and imaging features of oral squamous cell cancer in Serbia: a retrospective cross-sectional study

**DOI:** 10.1186/s12903-024-03869-8

**Published:** 2024-01-29

**Authors:** Aleksa Janović, Đurđa Bracanović, Svetlana Antić, Biljana Marković-Vasiljković

**Affiliations:** School of Dental Medicine, Center of Diagnostic Radiology, University of Belgrade, 6 Rankeova, Belgrade, 11000 Republic of Serbia

**Keywords:** Squamous cell Cancer, Oral cavity, Demography, X-Ray computed Tomography, Retrospective studies

## Abstract

**Background:**

The mortality of oral squamous cell cancer (OSCC) in Serbia increased in the last decade. Recent studies on the Serbian population focused mainly on the epidemiological aspect of OSCC. This study aimed to investigate the demographic and imaging features of OSCC in the Serbian population at the time of diagnosis.

**Methods:**

We retrospectively analyzed computed tomography (CT) images of 276 patients with OSCC diagnosed between 2017 and 2022. Age, gender, tumor site, tumor volume (CT-TV, in cm^3^), depth of invasion (CT-DOI, in mm), and bone invasion (CT-BI, in %) were evaluated. TNM status and tumor stage were also analyzed. All parameters were analyzed with appropriate statistical tests.

**Results:**

The mean age was 62.32 ± 11.39 and 63.25 ± 11.71 for males and females, respectively. Male to female ratio was 1.63:1. The tongue (36.2%), mouth floor (21.0%), and alveolar ridge (19.9%) were the most frequent sites of OSCC. There was a significant gender-related difference in OSCC distribution between oral cavity subsites (Z=-4.225; *p* < 0.001). Mean values of CT-TV in males (13.8 ± 21.5) and females (5.4 ± 6.8) were significantly different (t = 4.620; *p* < 0.001). CT-DOI also differed significantly (t = 4.621; *p* < 0.001) between males (14.4 ± 7.4) and females (10.7 ± 4.4). CT-BI was detected in 30.1%, the most common in the alveolar ridge OSCC. T2 tumor status (31.4%) and stage IVA (28.3%) were the most dominant at the time of diagnosis. Metastatic lymph nodes were detected in 41.1%.

**Conclusion:**

Our findings revealed significant gender-related differences in OSCC imaging features. The predominance of moderate and advanced tumor stages indicates a long time interval to the OSCC diagnosis.

## Background

Among all malignant tumors that may develop in the oral cavity, oral squamous cell cancer (OSCC) is the most common, accounting for more than 90% of cases [1]. It is the eighth most prevalent cancer type in Europe and the eleventh cancer-related cause of death [2]. OSCC has been attracting increased clinical and scientific attention in the last decade due to the frequent occurrence of nodal metastasis at the time of diagnosis and poor prognosis despite recent treatment improvements. Besides, OSCC is associated with a more significant deterioration of quality of life than other cancers [3]. Patients treated with radiotherapy (single or combined with surgical treatment) may suffer from significant side effects such as mucositis, opportunistic infections, impaired salivary function, radiation-induced caries, and osteoradionecrosis [4–6].

The major risk factors for the OSCC development are tobacco and alcohol consumption [7–11]. Chemical carcinogens from tobacco products and alcohol act synergistically in generating mutations in tumor suppressor genes and thus promoting carcinogenesis [12]. The OSCC typically arises in the mucosa of the oral cavity, such as the gingiva, oral tongue, and the floor of the mouth [7, 13]. It is more common among males with the peak incidence in the seventh decade of life [2, 7, 8]. Another type of OSCC is associated with the human papillomavirus (HPV) primarily with a persistent infection of high-risk oncogenic genotypes, such as HPV-16 and HPV-18 [12, 14]. The HPV-positive OSCC has a distinct pathway of carcinogenesis, resulting in a significantly different genetic tumor profile in comparison to the HPV-negative OSCC [12, 15]. The predilection sites for HPV-positive cancer are the oropharynx, tonsils, and the tongue base [16–18]. Patients with HPV-positive cancer are generally younger (peak incidence in the fifth decade of life) and have a better prognosis than HPV-negative OSCC [2, 7, 8]. HPV-positive and negative OSCCs also exhibit different imaging features. On computed tomography (CT) or magnetic resonance (MR) imaging, HPV-positive cancer presents as a well-defined exophytic lesion with vivid enhancement and cystic lymph node metastasis [19–21]. By contrast, HPV-negative OSCCs are often larger and ill-defined, with a propensity for adjacent muscle invasion and extra-nodal extension [19–21].

Global Cancer Estimates (GLOBOCAN) projections marked Serbia with the highest incidence and mortality of oral and pharyngeal cancer in Southern Europe, after Portugal and Croatia [2]. According to the Serbian National Cancer Registry data, oral and pharyngeal cancer became the eight most common cancer types in males [22–25]. Recent studies in the Serbian population focused on an epidemiological aspect of OSCC in a limited number of patients, including mortality/morbidity rate trends, the detection of risk factors, and the economic aspect of treatment [22, 24, 26–28]. One study also investigated the OSCC’s histopathological profile [29].

Cross-sectional imaging plays an integral role in the diagnostic workup of OSCC. It provides essential information on local tumor extent into soft tissue, regional spread to cervical lymph nodes, and bone invasion. These imaging features determine the tumor stage, the management strategy (single or multimodal therapy), the resectability assessment, and the extent of the surgical resection [30]. Recent changes in the TNM classification system introduced the depth of invasion (DOI) as a new parameter that became an integral part of the radiological OSCC assessment. Therefore, the current study aimed to investigate demographic and computed tomography (CT) imaging features of OSCC at the time of diagnosis in the Serbian population. Particular emphasis was to assess potential gender- and site-related differences in imaging features and stage of OSCC.

## Methods

### Study design and sample

**Fig. 1:**
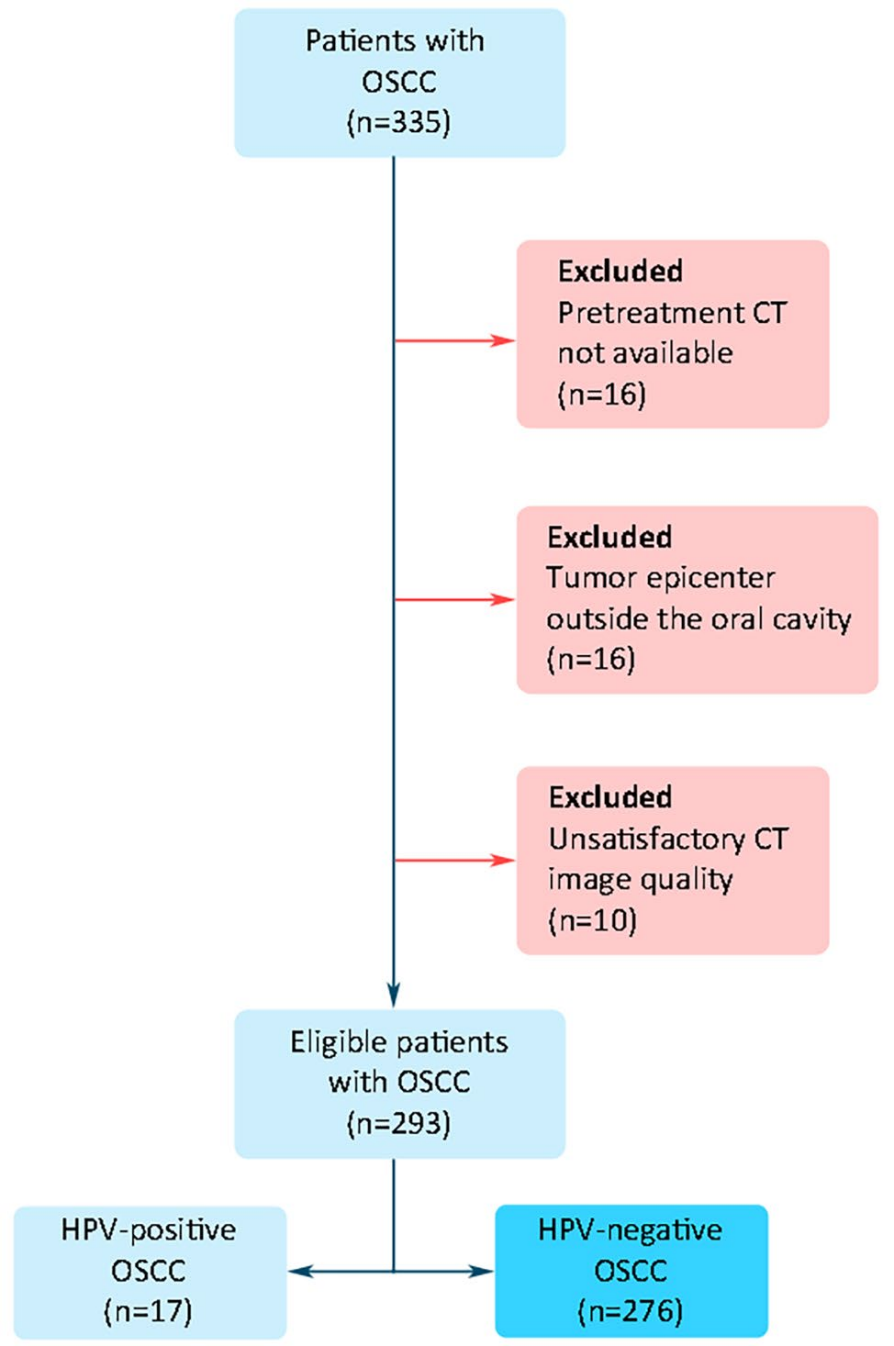
Flow chart of patient selection

The study retrospectively evaluated patients with primary squamous cell carcinoma in the oral cavity (excluding lips) diagnosed at the School of Dental Medicine, University of Belgrade, between January 2017 and December 2022. The patient selection protocol is illustrated in Fig. 1. Patients were excluded from the initial sample (*n* = 335) if (1) pretreatment CT was not available for analysis, (2) the tumor epicenter was located outside the oral cavity (e.g., in the tonsillar region or oropharynx), (3) quality of CT images was unsatisfactory (e.g., a significant part of the tumor covered with metal artifacts from tooth restorations). A total of 293 patients were considered eligible for the study. Patients’ medical records were then reviewed for the HPV status and divided into two groups (Fig. 1). HPV status was defined by immunohistochemistry staining of p16 protein during histopathological analysis. Due to a significantly lower number of patients with HPV-positive OSCC (*n* = 17), further analysis was performed on patients with HPV-negative OSCC (referred to as OSCC in the further text). Age and gender at the time of diagnosis were recorded for each patient. The distribution of OSCC according to gender and age category was calculated.

The study followed the ethical standards of the institutional and national research committee and the 1964 Helsinki Declaration and its later amendments or comparable ethical standards. Approval was obtained from the local Ethical Committee (Decision No. 36/33).

### CT analysis

OSCC imaging features were analyzed on initial pretreatment computed tomography (CT) images. CT examination was performed as a part of the standard diagnostic workup using a Philips Ingenuity Core 64-raw CT device (Philips Medical Systems, Cleveland, USA). Scanning was done in a supine position. Various maneuvers were applied during scanning (e.g., puffed cheek technique) to allow precise tumor detection and to avoid metal artifacts from tooth restorations. The neck was scanned from the skull base to the thoracic inlet before and after contrast administration. Acquired raw CT data were reformatted at the slice thickness of 1.0 mm. Obtained CT images of all patients were stored in DICOM format in the internal memory of the Picture Archiving and Communication System (Carestream Health, Rochester, NY).

Both soft tissue and bone window CT images were reviewed for the exact tumor location, tumor volume calculation (CT-TV, in cm^3^), depth of invasion (CT-DOI, in mm), and bone invasion (CT-BI, in %). Tumor location was recorded at the following anatomical subsites in the oral cavity: (1) alveolar ridge, (2) buccal mucosa and fornices, (3) retromolar trigone, (4) oral tongue, (5) floor of the mouth and (6) palatal mucosa. CT-TV was calculated in Carestream software for volume measurement by segmentation technique and automatic volume calculation on contrast-enhanced CT (CECT) images in soft tissue settings. CT-DOI was measured at the tumor epicenter, as illustrated in Fig. 1, from the mucosal surface to the deepest point of the tumor [31]. CT-BI was assessed in bone window CT images. CT-BI was marked as present if there was a visible erosion or osteolytic lesion on the jaw bone surface in direct contact with the tumor. Data on the local tumor extent, regional nodal spread, and distant metastases were also analyzed according to the 8th edition of the American Joint Committee on Cancer Staging of the oral cavity [32].

**Fig. 2:**
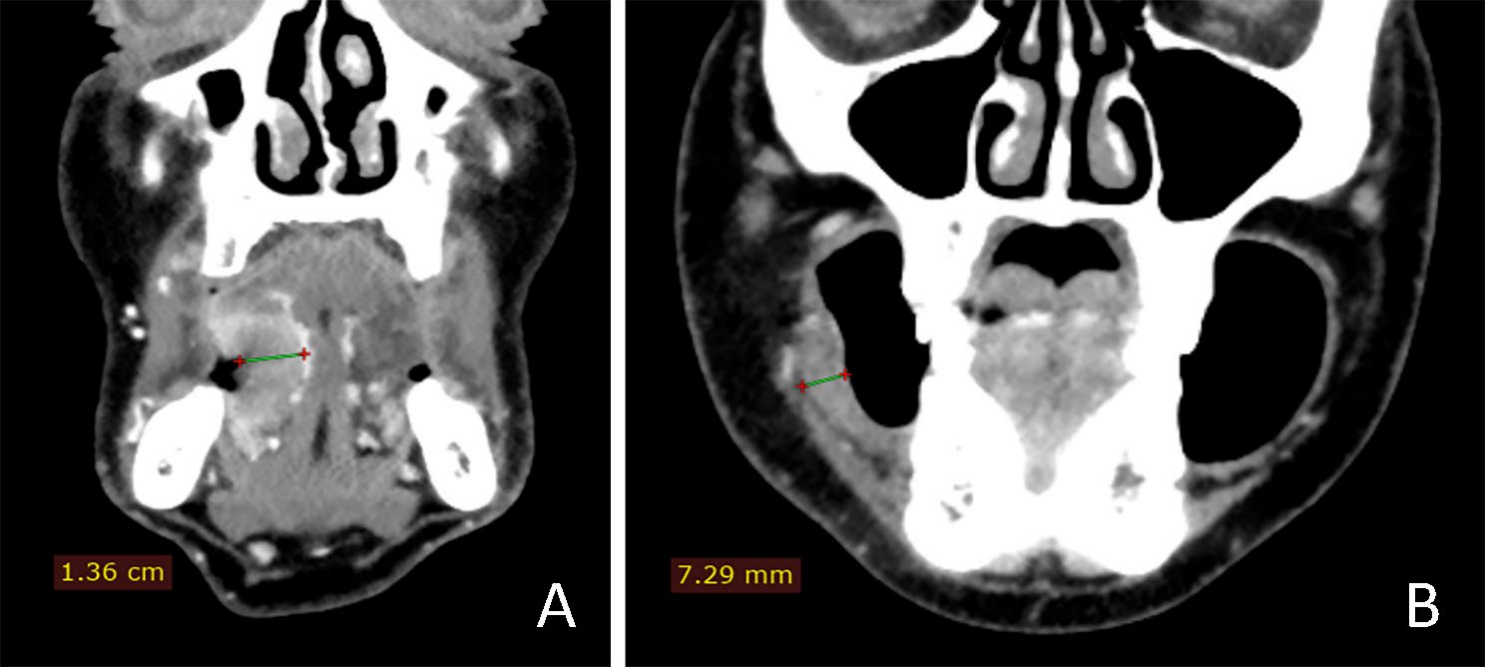
(**A**) Depth of tumor invasion (DOI) measured on the coronal reformatted contrast-enhanced CT image of the tongue OSCC. The tumor presented as a postcontrast hyperdense soft tissue mass. (**B**) DOI measured in the OSCC of the buccal mucosa. The tumor presented as an irregular thickening of the buccal and inferior fornix mucosa

### Statistical analysis

Statistical analysis was carried out using SPSS statistical software (version 15.0, Inc., Chicago, IL) [33, 34]. Parametric data were presented as mean ± standard deviation. The Kolmogorov-Smirnov test was applied to assess the normality of data distribution. Nonparametric data were presented as frequencies. Differences in age, CT-TV, and CT-DOI between genders were assessed by the Student’s t-test. ANOVA tested the difference in patient age between different tumor locations. Mann-Whitney test estimated gender-related differences in OSCC distribution, TNM status, and tumor stage. The statistical significance was set at 0.05.

## Results

The distribution of OSCC in 276 patients according to age, gender, and the oral cavity subsite is presented in Table 1. OSCC was more frequently diagnosed in males (61.9%) than in females (38.1%) with a male-to-female ratio of 1.63:1. Mean age ± standard deviation of mean (SD) was 62.32 ± 11.39 and 63.25 ± 11.71 for males and females, respectively, with no statistically significant difference (t=-0.649; *p* = 0.517). The peak incidence of OSCC was recorded in the seventh decade of life in both genders. In nine patients, OSCC occurred before 40 years. The youngest patient in our series was 30 years-old male.

**Table 1:** Distribution of OSCC according to gender, age, and subsite in the oral cavity

Feature	Male	Female	Total
**Number of cases**	171 (61.9%)	105 (38.1%)	276
Age*	62.32 ± 11.39	63.25 ± 11.71	62.67 ± 11.50
**Age category**			
**< 40**	5 (2.9%)	4 (3.8%)	9 (3.3%)
**41–50**	24 (14.0%)	9 (8.6%)	33 (11.9%)
**51–60**	38 (22.2%)	28 (26.7%)	66 (23.9%)
**61–70**	**62 (36.3%)**	**35 (33.3%)**	**97 (35.1%)**
**71–80**	32 (18.7%)	25 (23.8%)	57 (20.7%)
**> 81**	10 (5.9%)	4 (3.8%)	14 (5.1%)
**Subsite**			
**Alveolar ridge**	27 (15.8%)	28 (26.7%)	55 (19.9%)
**Buccal mucosa, fornices**	13 (7.6%)	25 (23.8%)	38 (13.8%)
**Retromolar trigone**	12 (7.0%)	8 (7.6%)	20 (7.3%)
**Tongue**	**71 (41.5%)**	**29 (27.6%)**	**100 (36.2%)**
**Mouth floor**	45 (26.3%)	13 (12.4%)	58 (21.0%)
**Palatal mucosa**	3 (1.8%)	2 (1.9%)	5 (1.8%)

* - presented as mean ± standard deviation

For the entire sample, the tongue (36.2%) was the most frequent subsite of OSCC, followed by the floor of the mouth (21.0%) and alveolar ridge (19.9%) in descending order (Table 1). The same subsite distribution of OSCC was recorded in males. In females, the tongue and alveolar ridge were also the two most common sites of OSCC, while the third most prevalent site was buccal mucosa, including fornices. Gender-related difference in the distribution of OSCC was statistically significant (Z=-4.225; *p* < 0.001). Patients with alveolar ridge OSCC had the highest mean age of 67.42 ± 10.01, whereas patients with tongue OSCC had the lowest mean age of 59.14 ± 11.74. When compared, this difference was confirmed as statistically significant (ANOVA − 8,278; *p* < 0.001). The mean age of patients having OSCC at other subsites was between these two values without significant intergroup differences.

**Fig. 3:**
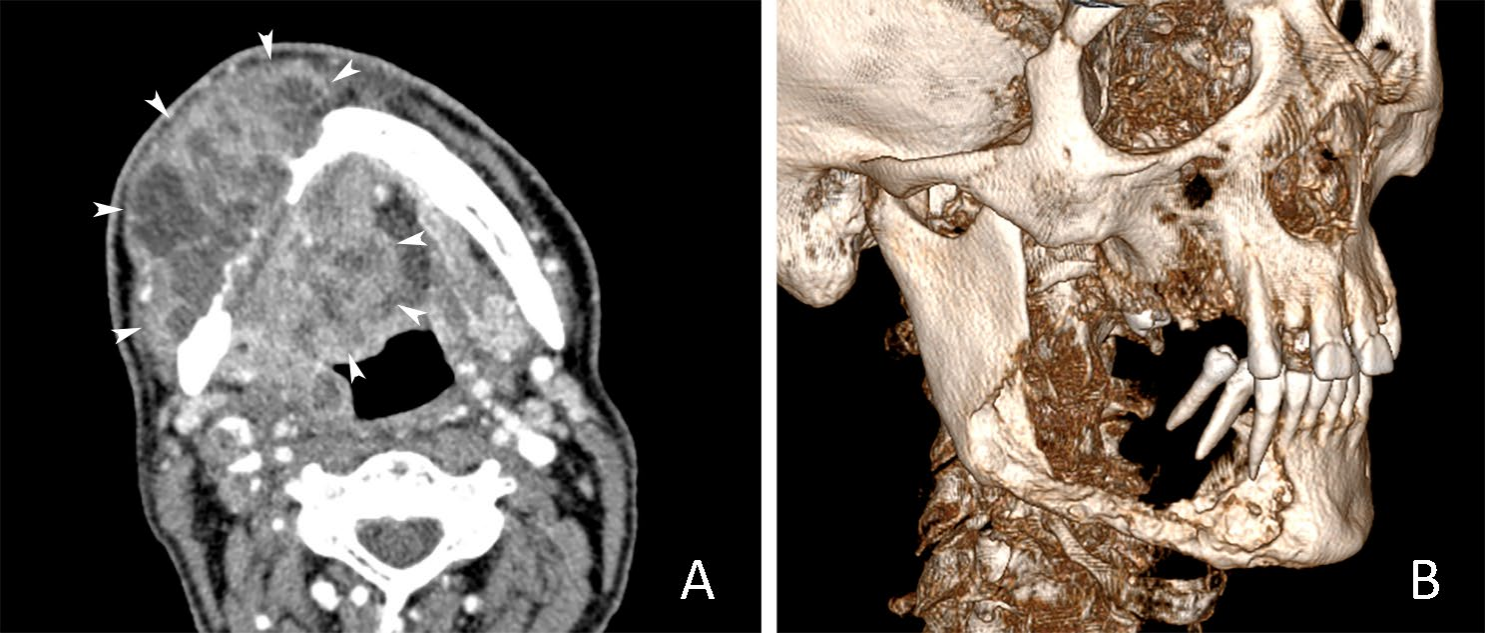
Large OSCC originating from the right alveolar ridge. (**A**) Axial CECT image in soft tissue window shows large heterogenous tumor mass with extensive local invasion of the surrounding soft tissues (white arrowheads depict tumor borders). Note the extensive bone destruction of the right mandibular body. (**B**) Volume rendering CT image shows the extent of bone destruction of the right mandibular body and angle

**Fig. 4:**
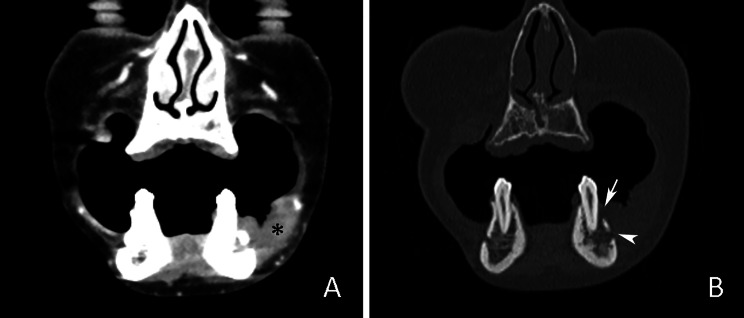
OSCC of the left buccal and inferior fornix mucosa. (**A**) Hyperdense tumor mass presented as an irregular mucosal thickening (black asterisk) on coronal CECT image in soft tissue window. (**B**) Signs of mandibular bone invasion on coronal CECT image in the bone window (white arrow shows destruction of the buccal cortical bone; arrowhead shows mental foramen). Note the disruption of the trabecular bone architecture and blurring of the cortical bone margins on the left

On CT images, OSCC typically presented as thickening of the oral mucosa or irregularly shaped soft-tissue mass, usually with intensive and homogeneous contrast enhancement (Fig. 2). Table 2 displays the estimated CT parameters of OSCC in relation to tumor localization. The highest mean CT-TV was recorded in the alveolar ridge OSCC (Fig. 3). Comparison between mean CT-TV in males (13.8 ± 21.5) and females (5.4 ± 6.8) revealed statistically significant differences (t = 4.620; *p* < 0.001). CT-DOI also showed a slight intersite difference. Except for palatal mucosa, the mean CT-DOI was higher than 10 mm at other subsites. There were also differences in mean CT-DOI between males (14.4 ± 7.4) and females (10.7 ± 4.4), which were statistically significant (t = 4.621; *p* < 0.001). Positive CT-BI was the most frequently detected at subsites closest to the jawbone surface, such as the alveolar ridge (72.7%), retromolar trigone (50.0%), palate (40.0%), and buccal mucosa and fornices (34.2%) (Fig. 4). CT-BI was detected in both gender groups without a statistical difference (Chi-Square 3.017; *p* = 0.08).

**Table 2:** CT features of the OSCC in relation to tumor location in the oral cavity

Subsite	CT-TV* (in cm^3^)	CT-DOI* (in mm)	CT-BI n (%)**
**Alveolar ridge**	16.4 ± 30.4	12.4 ± 6.9	40 (72.7)
**Buccal mucosa, fornices**	9.9 ± 15.6	11.9 ± 5.8	13 (34.2)
**Retromolar trigone**	4.6 ± 5.2	10.4 ± 4.3	10 (50.0)
**Tongue**	13.7 ± 25.7	12.9 ± 7.2	5 (20.0)
**Mouth floor**	13.3 ± 17.9	14.2 ± 7.2	13 (22.4)
**Palatal mucosa**	2.9 ± 1.5	9.3 ± 4.9	2 (40.0)

* - presented as mean ± standard deviation; ** - the percentage was calculated in relation to the total number of cases in each subsite.

Table 3 shows the distribution of OSCC according to the 8th Edition TNM classification and staging system. Most patients had T2 status of OSCC (31.4%) at diagnosis. T3 and T4a OSCC were the second most common stages for males and females, respectively. In general, two-thirds of patients were diagnosed with T3 or higher OSCC stage regardless of the gender subgroup. There were no statistically significant differences in T status between gender subgroups (Z=-1.929; *p* = 0.054). Almost every second patient had a tumor spread into the cervical lymph nodes (41.1%). Females most frequently presented with a single metastatic lymph node (N1) in contrast to males, who commonly presented with multiple lymph node metastases (N2b) (Table 3). This difference in N status was statistically significant (Z=-2.568; *p* = 0.010). The least frequent were N3a and N3b categories. None of the patients had distant metastasis (M1) at diagnosis. Considering the tumor stage, the most prevalent was the IVA stage in the entire sample (28.3%) and gender subgroups (28.7% in males and 27.6% in females). There were no statistically significant gender differences in the tumor stage (Z=-1.747; *p* = 0.081).

**Table 3:** TNM status and stage of OSCC at presentation in relation to gender

	Male n (%)	Female n (%)	Total n (%)
**T status**			
***T1***	11 (6.4)	14 (13.3)	25 (9.1)
***T2***	51 (29.8)	36 (34.3)	87 (31.4)
***T3***	48 (28.1)	21 (20.0)	69 (25.0)
***T4a***	46 (26.9)	32 (30.5)	78 (28.3)
***T4b***	15 (8.8)	2 (1.9)	17 (6.2)
**N status**			
***N0***	91 (53.2)	70 (66.7)	161 (58.3)
***N1***	29 (16.9)	18 (17.1)	47 (17.0)
***N2a***	12 (7.1)	6 (5.7)	18 (6.5)
***N2b***	32 (18.7)	9 (8.6)	41 (14.9)
***N2c***	5 (2.9)	2 (1.9)	7 (2.5)
***N3a***	1 (0.6)	0 (0)	1 (0.4)
***N3b***	1 (0.6)	0 (0)	1 (0.4)
**Stage**			
***I***	11 (6.4)	10 (9.5)	21 (7.6)
***II***	38 (22.2)	27 (25.7)	65 (23.6)
***III***	42 (24.6)	28 (26.7)	70 (25.4)
***IVA***	49 (28.7)	29 (27.6)	78 (28.3)
***IVB***	31 (18.1)	11 (10.5)	42 (15.1)

## Discussion

The global incidence of OSCC has been increasing. According to recent epidemiological studies, the annual incidence of OSCC increased from 355.000 newly diagnosed cases in 2018 to 377,713 cases in 2020 [13, 35, 36]. The number of OSCC cases has also been increasing in the Serbian population in the last few years [22–24]. According to the Serbian National Cancer Registry data, the age-standardized incidence rate increased from 0.34 in 2010 to 0.37 in 2018 [25]. In our study, the peak incidence of OSCC was recorded in the seventh decade of life. This could be partially attributed to the old age of the study participants. The highest number of patients with OSCC was diagnosed in the category of 61–70 years. There is an evident tendency for OSCC to affect younger adults. Almost one-third of patients were diagnosed in the fifth and sixth decade of life. The youngest patient in our series was 30 years-old male. Analysis of gender distribution demonstrated a predominance of males among OSCC cases, which followed global trends and previous studies [22, 28]. This finding might not be unexpected concerning increased exposure to risk factors highlighted in a recent national survey. The prevalence of tobacco and alcohol consumption in the Serbian population is generally high. In the national study of Pakovic et al., almost every third adult consumes alcohol, while more than 50% of study participants were former or current smokers [37]. Alcohol consumption was dominant among males, which could partly explain the higher prevalence of OSCC in this gender group. The fact that alcohol consumption has recently increased in the adolescent population is worrying [38]. Radovanovic et al. pointed out that more than 50% of adolescents consume alcohol, especially males in urban areas [38].

Concerning anatomical subsites in the oral cavity, tumor distribution differed significantly between genders. The tongue and mouth floor OSCC were dominant in males compared to females, for whom these locations accounted for only 40%. The second most common subsite of OSCC in females was the alveolar ridge. Comparing our results with the study of Dimitrijevic et al. [39] on the Serbian population between 1992 and 1995, the tongue is still the most prevalent site of OSCC. In general, a similar distribution was reported in international studies where the mouth floor and tongue were the most frequent sites of OSCC, accounting for 75% of all cases [1, 40, 41].

This study showed a wide discrepancy in CT-TV across anatomical subsites and at the single subsite, most prominently in the alveolar ridge OSCC. The later is reflected by relatively high standard deviation values. Similar significant discrepancies in TV values were reported in the literature, ranging up to 50 cm^3^ [42–46]. Additionally, in our sample, males presented with significantly larger mean CT-TV than females. From the surgical aspect, data about tumor size and volume are crucial prerequisites for planning a surgical approach. Kuznetsov et al. recently demonstrated the link between TV and resection margins [42]. The higher the tumor volume, the higher the risk for positive margins. The large OSCCs are often close to the critical anatomical structures, e.g., great vessels and nerves, which complicate the achievement of surgical clearance and thus lead to positive margins.

Besides the tumor size, the DOI of OSCC is a critical factor for tumor staging. The DOI is an integral part of the radiological and histopathological assessment of the OSCC. The 8th edition TNM classification system introduced DOI as a new tool for tumor stratification inside the T category (from T1 to T3) [32]. A growing body of literature suggests its importance as a prognostic factor for nodal involvement and overall and disease-specific survival rates in OSCC patients [47–51]. The DOI of 10 mm has been associated with a significant decline in the OSCC survival rate [50]. In our series, mean DOI was higher than the abovementioned threshold value at many subsites, suggesting that most of our patients may have already advanced diseases at the time of diagnosis and, consequently, poor prognosis. This statement was supported by the staging evaluation results, which indicate a significant number of patients in advanced stages of OSCCs (stages III and IV).

BI from OSCC is an additional important parameter that upgrades the tumor stage to a moderately advanced level (T4a), regardless of the tumor size, and thus may influence management [32]. Imaging is integral in detecting cortical BI, given that clinical examination cannot accurately predict it [52, 53]. CECT has priority for this purpose concerning its higher specificity (87–90%) compared to other imaging modalities, such as magnetic resonance imaging [54–56]. In general, the occurrence of BI varies between oral cavity subsites. BI is reported to be more common at the OSCC sites closest to the bone surface, e.g., alveolar ridge and retromolar trigone. The frequency of BI at these sites ranges up to 72% when compared with the tongue OSCC, which affects bone in less than 10% [54, 57]. In our series, intersite differences in BI showed similar ranges. As expected, BI was most commonly diagnosed in the alveolar ridge, retromolar trigone, and palatal OSCC. Despite significantly higher TV and DOI of OSCC in males, BI did not show significant gender differences. However, the fact that around 30% of our patients had CT signs of BI at presentation is concerning. This means that every third patient will be subjected to more complicated surgical management, e.g., marginal or segmental mandibulectomy/maxillectomy, depending on the degree of bone invasion. This may additionally influence the treatment outcome and quality of life, given that more extensive bone removal, such as in segmental mandibulectomy, often results in functional and cosmetic disturbances [3].

Lymphatic dissemination of OSCC typically occurs in the submandibular and upper jugular lymph nodes on the ipsilateral side [1, 49]. The reported incidence of lymph node metastases in the literature ranges between 6% and 45% [58]. Concerning N status in our sample, 41% of patients presented initially with lymph node metastasis. This is an essential finding, bearing in mind that the affection of the lymph nodes has a significant negative impact on a treatment plan, prognosis, and survival rate [1, 59]. It is the most important independent prognostic factor that reduces survival rate by 50% [30]. The rate of lymphatic metastases of OSCC differs due to tumor location and the local lymphovascular network [1]. In general, OSCC of the retromolar trigone and the mouth floor have a higher propensity to disseminate into neck lymphatics than other OSCC [1]. In our series, positive lymph nodes were most frequently diagnosed in the mouth floor and buccal mucosa OSCC with 46.6% and 42.1%, respectively. The percentage of positive lymph nodes in the retromolar trigone OSCC was similar to tongue OSCC (40%).

The fact that a significant number of our patients, particularly males, were diagnosed in the advanced stage of OSCC with lymph node involvement might have a negative impact on treatment success, prognosis, and survival. Our results undoubtedly indicate an evident long time interval to the diagnosis of OSCC that could partially explain increased mortality. Generally, the main factors that have been recognized to contribute to the long time interval to diagnosis are the lack of awareness of early signs and symptoms of OSCC and the absence of screening [60]. Healthcare practitioners at the primary healthcare level, such as specialists in general medicine and dentists, may play a crucial role in patient education and the early detection of the OSCC. More attention should be paid to patient education about the adverse effects of tobacco and alcohol abuse, particularly in adolescents and young adults. Oral mucosa is freely assessable to direct visualization on physical examination. Patients should be encouraged and educated to perform a self-examination, particularly males and those at a higher risk for developing OSCC. On the other hand, our findings should raise awareness about OSCC among health professionals and encourage them to participate actively in the prevention and early detection of OSCC. This study strongly suggests the need for planning and developing prevention strategies at the national level.

This study could be limited because data were collected from a single tertiary medical center and, therefore, might not be representative enough for the whole country. However, the study sample was significantly larger than in previous national studies and derived from the referent institution to diagnose and treat OSCC. Habits of study participants related to tobacco and alcohol consumption were not included due to limited or missing data in some patients. In order to fill this gap, we discussed our results in the context of recent national studies that reported the prevalence of smoking and alcohol consumption in the Serbian population. Another limitation relates to CT and metal artifacts from dental restorations that may decrease the quality or obscure the region of interest entirely. This was avoided by applying various dynamic examination techniques during scanning, such as the distension technique (Fig. 1). With this technique, tumors in the mucosa in the buccal region and the upper and lower fornix region can be better visualized, and DOI can be accurately measured.

## Conclusions

This study revealed several significant findings related to demographic and imaging features of HPV-negative OSCC that contribute to a better insight into the burden of this malignant tumor in the Serbian population. There was an evident shift in the OSCC incidence toward young adults. Gender-related differences in imaging features of OSCC were reflected in the significantly higher TV, higher DOI, and a higher number of affected cervical lymph nodes in male patients. The most concerning findings were a predominance of moderate and advanced tumor stages, bone invasion in every third patient, and frequent lymph node metastases at the time of diagnosis that suggest limited treatment options and unfavorable prognosis in most patients with OSCC.

## Data Availability

The datasets generated during and/or analyzed during the current study are not publicly available in order to maintain subject confidentiality. Data are available from the corresponding author only upon on reasonable request (Aleksa Janovic, email: aleksa.janovic@stomf.bg.ac.rs).
